# Gas6 enhances axonal ensheathment by MBP^+^ membranous processes in human DRG/OL promyelinating co-cultures

**DOI:** 10.1042/AN20130022

**Published:** 2014-01-24

**Authors:** Kathleen N. O’Guin, Ross C. Gruber, Cedric S. Raine, Hillary M. Guzik, Bradford K. Poulos, Bridget Shafit-Zagardo

**Affiliations:** *Department of Pathology, Albert Einstein College of Medicine, 1300 Morris Park Avenue Bronx, NY 10461, U.S.A.; †Analytical Imaging Facility, Albert Einstein College of Medicine, 1300 Morris Park Avenue Bronx, NY 10461, U.S.A.

**Keywords:** human OPC/DRG co-culture, myelination, oligodendrocyte, growth arrest-specific protein 6, BDNF, brain-derived neurotrophic factor, CNS, central nervous system, CSF, cerebral spinal fluid, DRG, dorsal root ganglia, ERK, extracellular-signal-regulated kinase, Gas6, growth-arrest-specific protein 6, IGF1, insulin-like growth factor 1, mAb, monoclonal antibody, MAG, myelin-associated glycoprotein, MAPK, mitogen-activated protein kinase, MBP^+^, myelin basic protein-positive, NB, neurobasal, NF, neurofilament, NGF, nerve growth factor, OL, oligodendrocyte, OPC, oligodendrocyte progenitor cell, pAb, polyclonal antibody, PI3K, phosphoinositide 3-kinase, T3, tri-iodothyronine, T4, thyroxine, TAM, Tyro3, Axl, and Mer

## Abstract

The molecular requirements for human myelination are incompletely defined, and further study is needed to fully understand the cellular mechanisms involved during development and in demyelinating diseases. We have established a human co-culture model to study myelination. Our earlier observations showed that addition of human γ-carboxylated growth-arrest-specific protein 6 (Gas6) to human oligodendrocyte progenitor cell (OPC) cultures enhanced their survival and maturation. Therefore, we explored the effect of Gas6 in co-cultures of enriched OPCs plated on axons of human fetal dorsal root ganglia explant. Gas6 significantly enhanced the number of myelin basic protein-positive (MBP^+^) oligodendrocytes with membranous processes parallel with and ensheathing axons relative to co-cultures maintained in defined medium only for 14 days. Gas6 did not increase the overall number of MBP^+^ oligodendrocytes/culture; however, it significantly increased the length of MBP^+^ oligodendrocyte processes in contact with and wrapping axons. Multiple oligodendrocytes were in contact with a single axon, and several processes from one oligodendrocyte made contact with one or multiple axons. Electron microscopy supported confocal Z-series microscopy demonstrating axonal ensheathment by MBP^+^ oligodendrocyte membranous processes in Gas6-treated co-cultures. Contacts between the axonal and oligodendrocyte membranes were evident and multiple wraps of oligodendrocyte membrane around the axon were visible supporting a model system in which to study events in human myelination and aspects of non-compact myelin formation.

## INTRODUCTION

Myelination is essential for efficient ensheathment and insulation of axons allowing for enhanced axonal conductance and transport. While a great deal of our understanding of myelination comes from studying rodent model systems, there is an incomplete understanding of how efficient myelination is achieved during human development, and reparative processes occurring subsequent to neurologic diseases in the central nervous system (CNS). This lack of knowledge has hampered our understanding of how to optimize treatment for humans. Unlike rodent oligodendrocytes, human oligodendrocytes do not synthesize mature myelin proteins in the absence of axons. Several studies show that additional factors that enhance survival, maturation and myelination in human-derived models are required. Our goal was to combine growth factors that enhance oligodendrocyte progenitor cell (OPC) survival and maturation to generate a human co-culture model that expresses myelin-synthesizing proteins resulting in myelination. Our earlier studies determined that a vitamin K-dependent protein, growth-arrest-specific protein 6 (Gas6), is a survival/maturation factor for human O4^+^ OPCs. When maintained in platelet-derived growth factor (PDGF)-containing medium in the absence of Gas6 there was significant apoptosis 6 days post-plating. The addition of 5.6 nM recombinant human Gas6 prolonged survival and promoted the expression of 2’,3’-Cyclic-nucleotide 3’-phosphodiesterase (CNPase) protein (Shankar et al., 2003, 2006).

Gas6, detected in cerebral spinal fluid (CSF) (Sainaghi et al., 2008, 2013), and expressed in neurons and stem cells, can signal through the mitogen-activated protein kinase (MAPK)/extracellular-signal-regulated kinase (ERK) and phosphoinositide 3-kinase (PI3K)/Akt pathways. In the nervous system, Gas6 is expressed and secreted by several types of neurons including motor neurons in spinal cord and large neurons of the dorsal root ganglion (DRG) (Li et al., 1996; Allen et al. 1999). Gas6 and its receptors are expressed widely in the CNS, and the interaction between Gas6 and its receptors have physiologically relevant function (Crosier and Crosier, 1997; Avanzi et al., 1998; Allen et al., 1999; Prieto et al., 1999, 2000; Tsiperson et al., 2010; Binder et al., 2011; Weinger et al., 2011).

Gas6 is a ligand for the Tyro3, Axl, and Mer (TAM) family of receptor tyrosine kinase. The relative affinity of Gas6 for its receptors is Axl>Tyro3>Mer. *In situ* hybridization studies performed in rat CNS demonstrated that members of the TAM family are expressed on neurons and in white matter during myelination (Allen et al., 1999; Prieto et al., 2000). Transcription of Axl and Tyro3 are increased in the presence of nerve growth factor (NGF), the growth factor essential for healthy axonal outgrowth (Gundersen and Barrett, 1979). RT–PCR studies showed that Axl, Tyro3, and Mer are expressed in human O4^+^ oligodendrocytes (OLs), cultured fetal human microglia, and all three RNAs are expressed in human fetal brain and spinal cord (Shankar et al., 2003), and in the rodent CNS (Prieto et al., 2000). We proposed that Gas6 ligand receptor interaction enhances signaling between axons and OLs during myelination. By combining growth factors that enhance survival and maturation of human OLs and neurons we have generated a human co-culture system that expresses mature myelin proteins and can be further manipulated for a more complete understanding of human myelination.

## MATERIALS AND METHODS

Human fetal tissue including spinal cord, DRG and brain were obtained from the Human Fetal Tissue Repository as approved by the Institutional Review Board of Albert Einstein College of Medicine and state and federal laws.

### DRG explant cultures

DRGs were prepared from specimens ≥15 gw. DRGs were stripped of meninges, placed on previously prepared coverslips (12 mm) coated with diluted (1:7) growth factor-reduced Matrigel (356230; BD Biosciences) and covered with 300 μl of NB (neurobasal)/F medium. NB/F consists of NB medium containing 2% B27 (Gibco), 4 mg/ml D-glucose (Sigma), 2 mM L-glutamine (Gibco), 1% antibiotic/antimycotic (Gibco), 50 ng/ml NGF (Harlan) supplemented with 10 μM 5-fluorodioxyuridine and uridine (Sigma) (Chan et al., 2004; Taveggia et al., 2008; Wang et al., 2012). Cultures were maintained at 37°C in 5% CO_2._ Every 3 days, the medium was alternated with NB medium, NBF medium minus 5-fluorodioxyuridine and uridine. After three alternate feeds in NBF and NB, the DRGs were maintained in NB until axons radiating from the explant reached the edges of the coverslip. The total time frame was ~3 weeks. All DRG explant cultures selected for co-culture were of similar axonal density.

### Enrichment of OPCs

OPCs were prepared from mixed glial cultures of brain ≥16 gw. The fetal mixed glial culture was established from fetal brain tissue 3weeks after the DRGs were plated (Jana et al., 2007; Taveggia et al., 2008). Mixed glial cells were grown in poly-D-lysine-coated T-75 flasks in Dulbecco's modified Eagle's medium (DMEM)/F12 plus 10% FCS for 9 days. An initial 2 h shake-off at 240 rev./min was performed to remove the microglia and fresh medium was added. On day 11, an 18 h overnight shake-off at 180 rev./min was performed to remove the OPCs from the adherent monolayer. Prior to plating on the DRG explant the nonadherent OPCs from the shake-off were purified by two rounds of plating on uncoated tissue culture dishes for 1 h at 37°C, 5% CO_2,_ to further eliminate contaminating microglia, and plated on the DRG (see below).

Direct isolation of OPCs from human fetal brain mixed glial cultures was the best method for preserving the integrity of OPCs for co-culture. We did not find immunopanning to be better than the direct isolation of enriched OPCs. By reducing the amount of manipulation we observed an increase in the total number of viable OPCs, and minimized cell death. All lineage-committed OLs express β_IV_ tubulin (Terada et al., 2005). Immunohistochemical examination of enriched OPC cultures determined that ~95% of the cells were positive for PDGFαR, the transcription factor Olig2 and β_IV_ tubulin. There were ≤5% GFAP^+^ astrocytes and no detectable Iba1^+^ microglia.

### OPC/DRG co-cultures

Unless otherwise noted, enriched OPCs purified from mixed glial cultures were immediately plated without further culturing on to the DRG explant at 1×10^5^ cells/well in defined medium consisting of DMEM high-glucose medium (Gibco 11995) supplemented with 2% B27 (Gibco 17504), 1% N2 (Gibco 17502), 20 ng/ml recombinant human brain-derived neurotrophic factor (BDNF) (Peprotech), 20 ng/ml insulin-like growth factor 1 (IGF1) (eBioscience), 40 ng/ml tri-iodothyronine (T3), 40 ng/ml thyroxine (T4), 5 ng/ml NT3, 1% antibiotic/antimycotic, plus and minus recombinant human Gas6 ranging from 0.77–5.6 nM (55–400 ng/ml; Amgen). When co-cultures were maintained in PDGF-containing medium, BDNF and IGF1 were not added. When PDGF was removed from the medium, BDNF and IGF1 were added and referred to as defined medium. Co-cultures were maintained at 37°C, 5% CO_2_. The medium was changed three times/week for 14 days whereupon co-cultures were fixed and analyzed by immunofluorescent staining.

While maintaining the DRG explant cultures, the mixed glial cultures and the DRG-enriched OPC co-cultures is challenging and time consuming, the culture conditions could be optimized to obtain consistent results. On average each co-culture experiment study took ~7 weeks to complete, and an additional 2–3 weeks to perform immunostaining and data analysis. For our co-cultures, Matrigel was utilized as the support matrix throughout all the studies.

### Growing OPCs in culture

**Figure 1:**
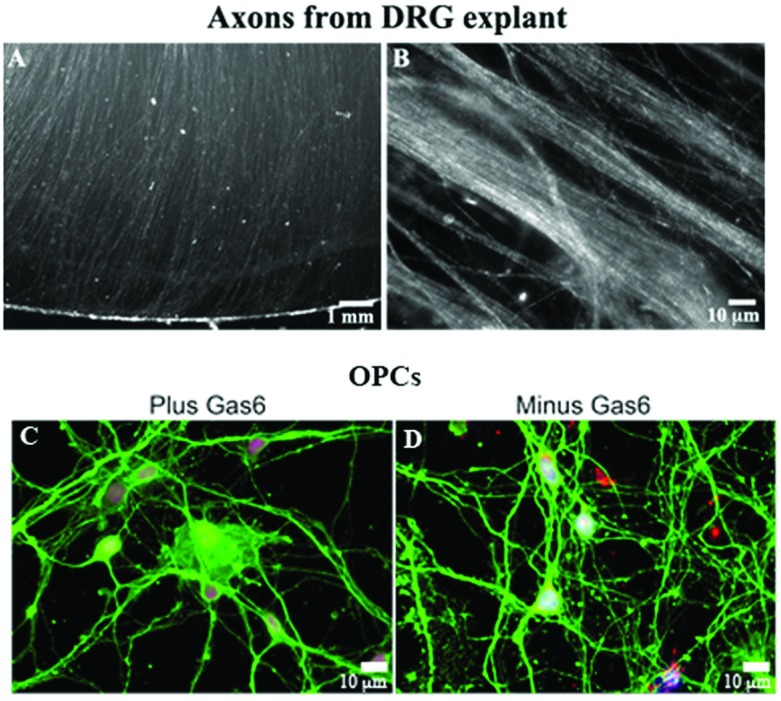
Generation of human fetal DRG explants (A and B) with parallel arrays of NF^+^ axons, and enriched OPCs from mixed glial cultures (C and D) (**A**) Axons radiating from a DRG explant maintained in culture for 3 weeks. (**B**) NF^+^-stained axons radiating from a DRG explant. (**C** and **D**) Enriched OPCs from human fetal brain maintained in the presence (**C**) and absence (**D**) of Gas6 are β_IV_ tubulin^+^ (green) and express Olig1 (red) in the nucleus following 20 days in culture. Blue is Hoechst stain. An Olympus digital microscope was used.

To determine the purity of the OPCs, enriched OPCs were isolated from mixed glial culture (Jana et al., 2007) and maintained in OPC medium [NB medium (Gibco 21103-049), DMEM high-glucose (Gibco 11995-065), 1 mM pyruvate (Gibco 11360-070), 5 μg/ml insulin (Sigma I6634), 10 μg/ml biotin (Sigma B4639), 0.1% Cellgrow Trace Elements B (MT99-175-C10), 2% B27 (Gibco 17504-044), 2 mM L-glutamine (Gibco 25030-081), 1% antibiotic/antimycotic (Gibco 15240-096), 100 μg/ml transferrin (Sigma T1147), 60 ng/ml progesterone (Sigma P8783), 100 μg/ml BSA (Sigma A4161), 16 μg/ml putrescine (Sigma P7505), 40 ng/ml sodium selenite (Sigma S5261), 40 ng/ml T3 (Sigma T6397), 40 ng/ml T4 (Sigma T1775)] at 37°C, 5% CO_2_, for 20 days, and then fixed in 4% paraformaldehyde. By immunohistochemistry, OPCs were stained for the OL markers β_IV_-specific tubulin and the transcription factor Olig1 (Figure 1). In addition, O4 and PDGF-Rα were consistently positive (data not shown).

### ELISA assay

Recombinant human Gas6 (Amgen) grown in vitamin K-containing medium was measured using the human Gas6 Quantikine ELISA kit (DGAS60, R&D Systems) following the manufacturer's protocol.

### Antibodies, TUNEL, immunofluorescent staining, microscopy, and measurement of the length of MBP ensheathment of axons

Myelin basic protein (MBP) monoclonal antibody (mAb) SMI99 or mAb SMI94 (1:500; Covance) was used to confirm myelination in co-cultures. Chick neurofilament (NF) polyclonal antibody (pAb) (1:5000; Millipore) was used to identify axons. Myelin-associated glycoprotein (MAG) antibody mAb 1567 was obtained from Millipore. Olig1 pAb was a gift from Dr John Alberts, Dana Farber Institute. Olig2 pAb (Dr John Alberts, and Millipore) were used to identify the percentage of Olig2^+^ oligodendrocytes that were MBP^+^, and in contact with axons. β_IV_ tubulin (1:400; Sigma) was used to identify OLs (Terada et al., 2005). Two PDGF-Rα pAbs, an anti-rabbit obtained from Dr William Stallcup and an anti-goat (R&D Systems), were used to demonstrate that the OPC cultures were PDGF-Rα-positive (data not shown). Co-cultures were incubated with antibodies against P0 (anti-chick, Millipore), and PMP22 (anti-rabbit, Sigma) to verify that the co-cultures were free of Schwann cell contamination (data not shown). P0 was generously provided by Dr James Salzer (NYU, NY).

Antibodies evaluated for nodal and paranodal proteins included (Caspr), obtained from Dr David Colman, neurofascin antibody L11A/41.6 mAb, Kv1 channel, ankyrinG (neuromab@ucdavis.edu), and several neurofascin antibodies obtained from Dr Barbara Zonta and Dr Peter Brophy (University of Edinburgh). Co-cultures were fixed in 4% paraformaldehyde in PBS, washed three times in 1×Tris-buffered saline, pH 7.4 (1×TBS), methanol-fixed for 10 min at −20°C, washed with 1×TBS, followed by a 1 h incubation in 2% goat serum and 5% non-fat dried milk in 1×TBS+0.1% Triton X-100. All antibodies were diluted in 5% non-fat dried milk in 1×TBS+0.1% Triton X-100, and incubated overnight at 4°C. Cultures were washed three times in 1×TBS+0.1% Triton X-100 and incubated with AlexaFluor-conjugated, isotype-specific secondary antibody for 1 h at room temperature (22°C). Sections were visualized on an Olympus Digital Microscope or a Zeiss Axioskop2 plus microscope with an AxioCam MRC camera. Randomly selected fields were selected on the MBP^+^ (green) channel, selecting MBP^+^ OLs with no knowledge of association with the NF^+^ axon (red). For some studies, additional Z-series images were collected by confocal microscopy using the Lecia SP2-AOBS. To measure the length of axonal ensheathment by MBP^+^ oligodendrocyte processes, multiple ×60 oil fields were photographed and the length of the processes were calculated in ImageJ (NIH). Additional measurements were obtained following determination of the longest MBP^+^ process length using Volocity 3D image analysis software. The software permits visualization of z-stacks as interactive 3D images that improve the resolution of confocal images. An *in situ* cell death detection kit fluorscein (Roche) was used to analyze TUNEL^+^ cells/×60 fields in co-cultures minus and plus Gas6 (*n*=3). Briefly, random ×60 fields from three coverslips/condition were viewed on the NF^+^ channel to avoid biasing the TUNEL^+^ fields. Merged images were analyzed for the mean number of condensed stained nuclei.

### Statistical analysis

Unless otherwise noted, statistical analysis was evaluated in GraphPad Prism using the unpaired *t* test. A Mann–Whitney test was performed to evaluate the length of the longest processes.

### Electron microscopy (EM)

Co-cultures maintained for 14 days in defined medium minus and plus Gas6 were fixed with 2.0% paraformaldehyde, 2.5% glutaraldehyde and 0.1 M cacodylate buffer (pH 7.4), and post-fixed with 1% osmium tetroxide followed by 1% uranyl acetate, dehydrated through a graded series of ethanol and embedded in LX112 resin (LADD Research Industries). Ultrathin (80 nm) sections were cut on a Reichert Ultracut UCT, stained with 0.01% uranyl acetate followed by lead citrate and viewed in a JEOL 1200EX or an Hitachi H600S instrument. Toluidine blue-stained 1 μm epoxy sections were examined by light microscopy (Tsiperson et al., 2010). Co-cultures in the absence of Gas6 were not processed further for EM as the MBP^+^ immunofluorescent staining indicated that ensheathment was sparse, short or incomplete and, therefore, requisite ensheathment necessary to observe wrapping and myelin by EM would not be observed.

## RESULTS

### Establishment of DRG/OL co-cultures using human fetal DRG explants and enriched OPCs purified from mixed glial cultures

This is the first report of successfully maintaining human fetal DRG in explant culture with long axons radiating from the explant and reaching the edge of the coverslip. We have determined that using growth-factor-reduced Matrigel stabilized the axons better than fibronectin or poly-lysine alone or in combination with Matrigel. Figure 1 shows a DRG explant with parallel arrays of axons by phase microscopy (Figure 1A), and axons following incubation with a chick NF polyclonal antibody (Figure 1B). In our experience, a 3 week DRG explant was optimal for use for co-culture resulting in axons that extended to the edge of the 12 mm coverslip. DRGs less than 15 gestational weeks could not support MBP^+^ OL expression in co-culture and were not included in analyses. Optimal co-cultures were obtained when the age of both the DRG and the OPCs were >16 gestational weeks.

Using our current method to isolate enriched OPCs from fetal brain, we examined whether addition of Gas6 (2.8 nM, 200 ng/ml) to β_IV_ tubulin^+^ OPCs (*n*=3 coverslips/treatment) would enhance OPC maturation following 20 days in culture. Fixed cultures were incubated with antibodies against β_IV_ tubulin (green) and the transcription factor Olig1 (red), and stained with Hoechst dye (blue). β_IV_ tubulin^+^ specific OLs with long bipolar and tripolar processes were observed in all OPC cultures, with Olig1 in the nucleus. Post-mitotic OLs express Olig1 in the nucleus, and during myelination Olig1 is expressed in the cytosol (Arnett et al., 2004). As shown in Figures 1(C) and 1(D), based on the β_IV_ tubulin and Olig1 staining there was no dramatic morphologic changes in the OPCs.

### Characterization of human DRG/OPC co-cultures supplemented with Gas6

Human OLs do not express mature myelin proteins in the absence of axons. Therefore, we tested whether different cocktails of growth factors and hormones in serum-free medium support myelination in human OL/DRG co-cultures. Since neurons synthesize and secrete Gas6, we measured the amount of Gas6 secreted in the co-culture medium. After feeding co-cultures in defined medium supplemented with 20 ng/ml IGF1 and BDNF for 3 days, the medium was analyzed for Gas6 using a human Gas6 ELISA kit. The amount of Gas6 in the spent medium was ~852 pg/ml or 12 pM. Several sources of recombinant Gas6 are commercially available; however, we have only used recombinant human Gas6 obtained from Dr Brian Varnum at Amgen. This full-length human Gas6 was grown in vitamin K-containing medium to ensure γ-carboxylation of Gas6 required for activation and phosphorylation of the TAM receptors. Using the ELISA assay we determined that the Gas6 stock was stable over time and equivalent to 1.86 mg/ml. In our co-cultures, we have examined a range of Gas6 concentrations from 0.35 nM to 5.6 nM (25 ng/ml to 400 ng/ml). We determined that medium supplemented with 0.77–5.6 nM Gas6 was beneficial for MBP^+^ process extension along the axon and enhancement of axonal wrapping. We have determined that 0.35 nM Gas6 was not effective, and concentrations greater than 2.8 nM were similar to the 1.4 and 2.8 nM doses.

**Figure 2:**
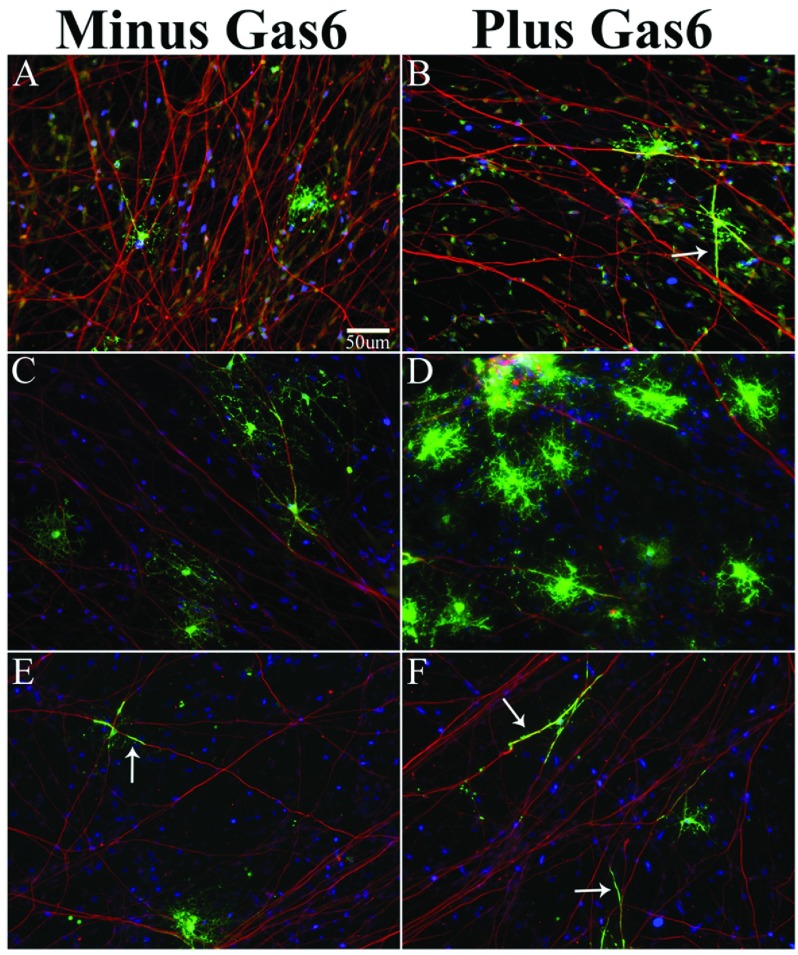
Human OPC/DRG co-cultures in medium containing PDGF+Gas6 express MBP^+^ processes that align and elongate along NF^+^ axons, while OLs in co-cultures maintained in BDNF+IGF1+Gas6 have more mature MBP^+^ processes that ensheath axons Immunofluorescent staining and confocal microscopy show MBP**^+^** OLs (green) and NF**^+^** axons (red; 1:2000; chick IgY-cy3) captured on an Olympus Digital microscope after 14 days in co-culture; scale bar for all images is 50 μm. (**A**) Co-cultures maintained in PDGF medium minus Gas6. (**B**) Co-cultures maintained in PDGF medium plus Gas6 have MBP^+^ OL processes aligned with axons. (**C** and **E**) Co-cultures maintained in IGF1+BDNF-defined medium minus Gas6. (**D** and **F**) Co-cultures maintained in IGF1+BDNF-defined medium plus Gas6. Scale bar in (**A**) designates magnification for all panels.

We evaluated human MBP^+^ OLs after 14 days in a differentiating medium characterized for rat co-cultures (Chan et al., 2004). Using that medium, as well as co-cultures maintained in medium with 10 ng/ml PDGF, we observed very few MBP^+^ OLs relative to the total number of Olig2^+^ OPCs. In PDGF-containing medium (*n*=6 co-cultures), there were 24.2±5.7 MBP^+^/Olig2^+^ oligodendrocytes per coverslip. In co-cultures with 2.8–5.6 nM Gas6 (*n*=4 co-cultures) there was no significant increase in the number of MBP^+^ oligodendrocytes and the total number of Olig2^+^ OPCs did not increase; *P*>0.05. The MBP^+^ OLs in co-culture plus or minus Gas6 was <0.5% of the total OPCs plated. As shown in Figure 2(A), analysis of MBP^+^ OLs (green) determined that the majority of the OLs maintained in PDGF medium had minimal to no interaction with the axon. Figure 2(B) shows that while the number of MBP^+^ OLs did not increase in the presence of Gas6 there were more MBP^+^ OL processes in direct contact with and parallel to axons (arrow) by immunofluorescent staining and light microscopy. The mean length±S.E.M. of the longest MBP^+^ linear segment was unchanged in the absence (8.6±5.3 μm) or presence (8.2±4.1 μm) of Gas6, indicating that in PDGF-containing media, Gas6 does not enhance MBP^+^ OL process outgrowth but does enhance MBP^+^ OL process association with the axon.

### Gas6 increases the number of OL processes ensheathing axons in co-culture maintained in IGF1+BDNF

Following addition of IGF1+BDNF to our defined medium the total number of MBP^+^ OLs/co-culture was significantly increased compared with co-cultures maintained in PDGF-containing medium. In the presence of IGF1+BDNF the mean number of MBP^+^ OLs/co-culture was 223.3±37.4 (*n*=6 co-cultures). In IGF1^+^BDNF^+^ medium supplemented with 0.77 nM Gas6 the mean number of MBP^+^ OLs/co-culture was 209±50.1 (*n*=6 co-cultures) with no significant difference in the overall number of MBP^+^ OLs (*P*>0.05); the addition of higher concentrations of Gas6 (2.8–5.6 nM) did not increase the overall number of MBP^+^ OLs. Co-cultures plus and minus Gas6 were stained for MBP, NF and TUNEL. The mean number of TUNEL^+^ cells±S.E.M./×60 fields was determined from three separate coverslips/condition. Quantification determined that there were fewer TUNEL^+^ cells in the Gas6-treated co-cultures (1.3±0.35/×60 field) relative to the cells in defined medium only (5.1±0.83/×60 field); *P*<0.02.

**Figure 3:**
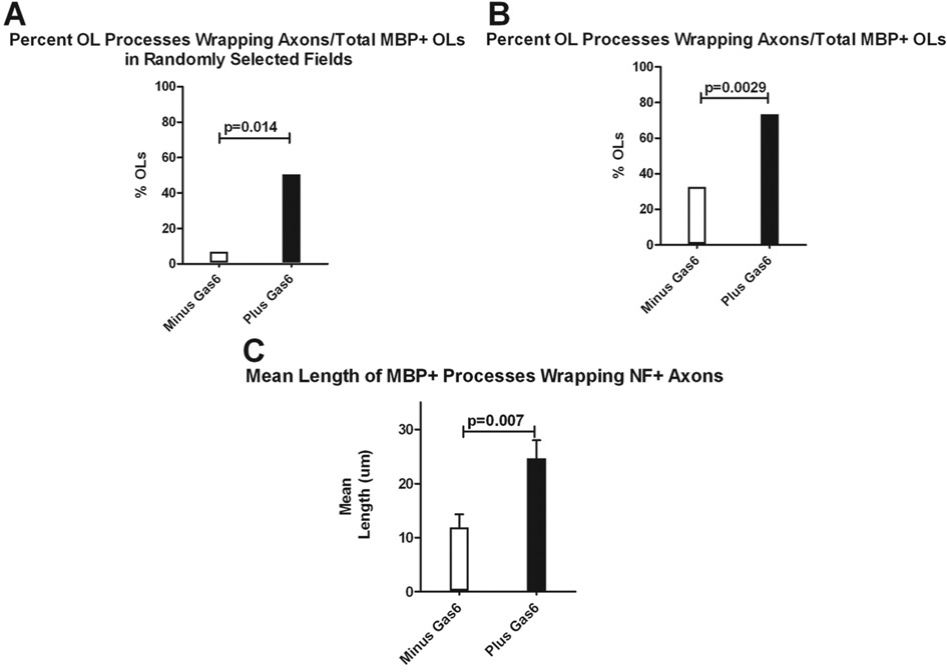
Gas6 enhances OL wrapping and mean process length in human co-cultures For experiments 1 and 2, co-cultures were maintained in defined medium as defined in the DRG/co-culture section of the Materials and methods (DMEM supplemented with 2% B27, 1% N2, 20 ng/ml rhBDNF, 20 ng/ml IGF1, 40 ng/ml T3, 40 ng/ml T4, 5ng/ml NT3, antibiotic/antimycotic, plus and minus Gas6). (**A**) Experiment 1, multiple, randomly selected MBP^+^ fields were photographed. From each random field, the percentage of MBP^+^ oligodendrocytes with processes wrapping axons relative to the total number of MBP^+^ oligodendrocytes/field was calculated. (**B**) Experiment 2, MBP^+^ OLs with obvious processes aligned with an axon were photographed, and the percentage of MBP^+^ OLs with processes wrapping axons relative to the total number of MBP^+^ OLs/field was calculated. (**C**) Histogram depicts the mean length (μm) of MBP^+^ processes wrapping NF^+^ axons in Gas6^−^ and Gas6^+^ co-cultures; *P*=0.007; Mann–Whitney test.

Figures 2(C)–2(F) show representative images from multiple co-cultures maintained in IGF+BDNF minus Gas6 (Figures 2C and 2E) and plus Gas6 (Figures 2D and 2F) with MBP^+^ OL processes wrapping axons (see arrows in Figures 2E and 2F). To test the prediction that Gas6 enhances MBP^+^ OL process alignment and membrane ensheathment of axons, co-cultures were treated with Gas6 for 14 days. The addition of Gas6 led to a significant increase in the number of OL processes ensheathing axons in several independent experiments (*P*<0.05). For each experiment, a minimum of three coverslips/treatment were immunostained for MBP and NF protein and ~20 randomly selected MBP^+^ oligodendrocytes were photographed by fluorescent microscopy (×60 oil). Using digital microscopy, one-dimensional analysis (length), and Image J, the percentage of MBP^+^ processes (green) wrapping NF^+^ axons (red) relative to the total number of MBP^+^ OLs/field was determined for each coverslip, and the mean value was calculated/treatment. We determined that Gas6, at 0.77 nM and 2.8 nM, led to a significant increase in the number of OL processes ensheathing axons (*P*<0.05). In the presence of Gas6 (*n*=5 coverslips) there was a ~6-fold increase in the number MBP^+^ oligodendrocyte processes aligned and wrapping axons relative to co-cultures maintained in the absence of Gas6 (*n*=4 coverslips). The histogram in Figure 3(A) shows that in the presence of Gas6 the percentage of wrapped axons in random fields was 50.5% while in the absence of Gas6 the percentage of wrapped axons was 6.7%; *P*=0.014. Although the overall percentages varied from experiment to experiment, we consistently observed significantly more wrapping in Gas6-containing co-cultures. In another experiment presented in Figure 3(B), we observed 73.3% of MBP^+^ oligodendrocytes ensheathed axons in cultures maintained in 0.77 nM Gas6 relative to 38.9% MBP^+^ OLs ensheathing axons in medium minus Gas6; *P*=0.0029. There was no difference in the mean total number of MBP^+^ OLs in the co-cultures plus and minus Gas6, *P*>0.05. In addition, using ImageJ we measured the length of the longest MBP^+^ OL process completely wrapping the axon. Figure 3(C) shows that the mean value of the longest MBP^+^ process wrapping an axon in the Gas6-containing co-cultures was 24.7 μm ±3.3 and the mean value for the co-cultures minus Gas6 was 11.9 μm ±2.4; *P*=0.007.

Having established that defined medium containing IGF1, BDNF and Gas6 was optimal for OL processes ensheathing axons we examined whether DRG explants isolated from the cervical, thoracic and lumbar levels of the cord (17–19 gestational weeks) supported the differentiation of OPCs to MBP^+^ oligodendrocytes. Co-cultures consisting of a DRG explant from each level of the cord and OPCs were analyzed for the total number of MBP^+^ oligodendrocytes/co-culture. The total number of MBP^+^ oligodendrocytes/co-culture plus and minus Gas6 was unchanged. The mean value was: cervical, 189 versus 153 MBP^+^ OLs/co-culture; thoracic, 297 versus 280 MBP^+^ OLs/co-culture; lumbar, 142 versus 115 MBP^+^ OLs/co-culture. Having demonstrated that DRGs from all levels support OPC maturation, we repeated this study and examined whether Gas6 (0.77 nM) enhanced the percentage of MBP^+^ OL processes that ensheath NF^+^ axons relative to defined medium minus Gas6. We photographed MBP^+^ OLs aligned with NF^+^ axons, and calculated the percentage of axonal processes ensheathed by MBP^+^ OL processes/40× field. The number of MBP^+^ OL processes wrapping at least one axon relative to the total number of MBP^+^ OL processes parallel to the axon was determined for all images. The percentage of MBP^+^ OLs wrapping axons in Gas6-containing medium relative to defined medium minus Gas6 was: cervical, 72% (±1.5; *n*=2) versus 35% (±1.0; *n*=2); thoracic, 72.5% (±8.5; *n*=5) versus 24.6% (±11.3; *n*=5; *P*=0.01); lumbar, 61% (±4.6; *n*=3) versus 25% (±7.1; *n*=3; *P*=0.013).

### Human co-cultures express MAG and MBP, but not P0

**Figure 4:**
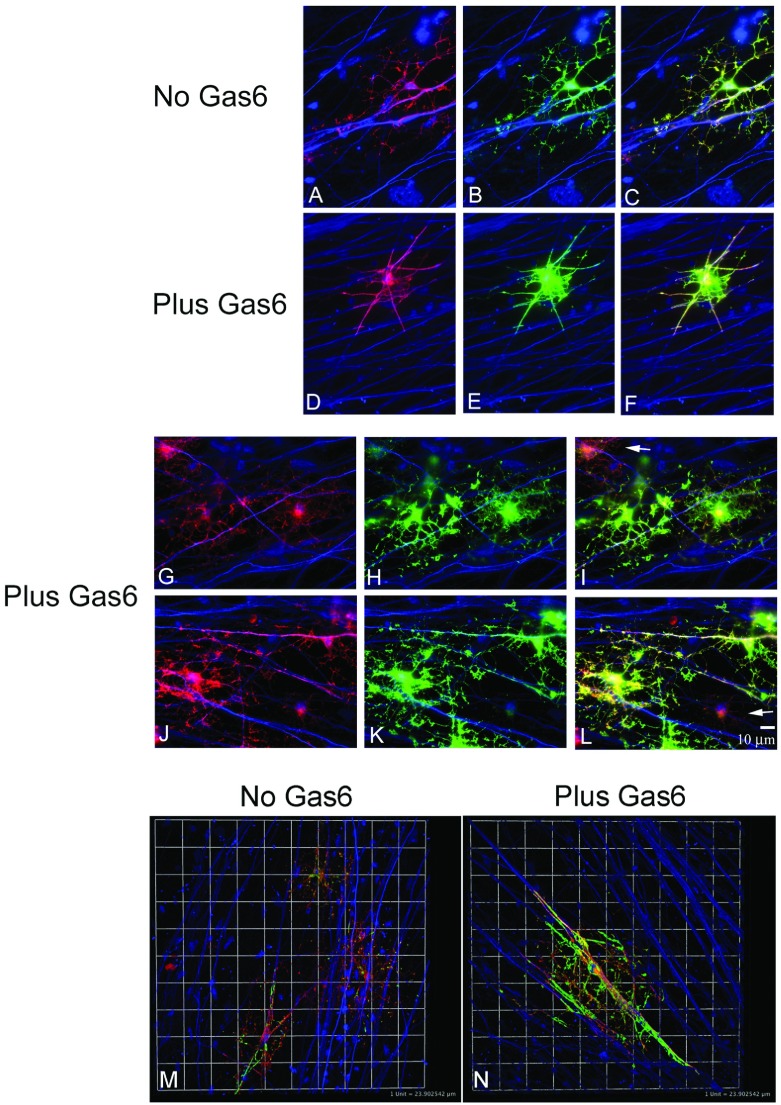
Multiple MAG^+^MBP^+^ OL processes from a single OL can ensheath the same axon and multiple axons Co-cultures were maintained in defined medium plus and minus Gas6 for 14 days (see the Materials and methods section). (**A**–**L**) Immunofluorescent staining and light microscopy. MAG^+^ (red), MBP^+^ (green) OLs, NF^+^ (blue) axons; scale bar=10 μm. (**M** and **N**) Z-series and confocal microscopy shows wrapping of MAG- and MBP-positive processes. Maximal projection of MBP^+^ processes was obtained in Volocity. Linear line of a box corresponds to 1 unit=24 μm. Backgrounds were adjusted to the negative primary antibody isotype controls. For example, the MAG primary antibody (IgG1) was substituted with primary antibody MOPC-31C purified clone for mouse IgG1 (BD Pharmingen; 557273).

To examine the expression of additional mature myelin proteins immunofluorescent staining on fixed co-cultures was performed using antibodies against P0, a PNS-specific marker, MAG, MBP, and NF. P0 expression was not detected in any co-cultures examined. Figure 4 shows that MAG^+^ (red) and MBP^+^ (green) OLs are expressed in IGF1^+^BDNF^+^ co-cultures. MAG^+^MBP^+^ OLs with extensively branched processes were observed both in the absence and presence of Gas6 (*n*=4 coverslips/condition; *P*>0.05). In the presence of Gas6, MAG^+^ processes (red, Figures 4G and 4J) and MBP^+^ processes (green, Figures 4H and 4K) wrapping NF^+^ axons (blue) are observed. In Figures 4I and 4L (arrows), MAG^+^ OLs that express little MBP immunoreactivity and have no processes wrapping axons are shown. OLs expressing MBP have more myelin membrane sheets associated with axon ensheathment (Figures 4H–4N). We examined MAG^+^MBP^+^ OLs by Z-series confocal microscopy and Volocity. All images were captured at the same laser intensity. Figure 4(M) shows three strongly MAG^+^ OLs from a co-culture grown in defined medium minus Gas6. The OL in the lower-left corner is strongly MBP^+^ and maximal projection indicates that the process is wrapping axons. Consistent with our data, OLs grown in defined medium plus Gas6 have more extensive wrapping. Figure 4(N) shows maximal projection of an OL with multiple MBP^+^ processes wrapping multiple axons. MAG expression appears to extend further to the periphery of OL processes suggesting that MAG expression precedes MBP in outer sheet formation of human fetal OLs (Figures 4M and 4N). Co-cultures maintained in the absence or presence of Gas6 had a similar percentage of OL processes with MAG extending farther than MBP in OL processes along the axon, 56% versus 54% respectively (*n*=4, *P*>0.05). Consistent with functional OLs, we found that a single OL could initiate wrapping of a single axon at multiple sites. Also, MBP^+^ OLs could wrap multiple axons.

**Figure 5:**
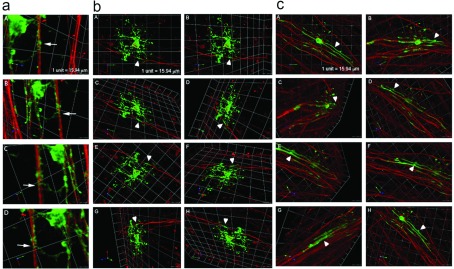
MBP^+^ OL processes (green) spiral and ensheath multiple NF^+^ axons (red) in co-cultures maintained for 14 days in defined medium minus (a and b) and plus (c) Gas6 Identification of multiple Z-series images captured by confocal microscopy (Leica) and analyzed in Volocity. The arrow in the image a shows an MBP^+^ OL process spirally wrapping the axon. Images in (**a** and **b**) show MBP^+^ OLs wrapping NF^+^ axons in co-cultures maintained in defined medium only. (**c**) A representative image from a Gas6-treated co-culture. Arrowheads in (**b** and **c**) indicate areas where MBP^+^ (green) membranes totally ensheath the axon (red).

To further explore if Gas6 consistently enhanced the ensheathment of axons by MBP^+^ OL processes, we evaluated MBP^+^ processes aligned with the axon for evidence of complete wrapping by Z-series confocal microscopy and 3D analysis. Figure 5(a) shows that in co-culture maintained in the absence of Gas6, MBP^+^ OLs processes were able to spirally wrap axons (arrow). Figure 5(b) shows an additional OL with MBP^+^ processes ensheathing the axon (arrowhead) (Supplementary Movie 1 at http://www.asnneuro.org/an/006/an006e135add.htm). Consistent with previous experiments, cultures minus Gas6 had fewer MBP^+^ OL processes wrapping axons, and the length of the longest wrap of an OL process completely around an axon was significantly shorter. Figure 5(c) shows the Volocity projection of the longest process observed in the co-culture in defined medium plus Gas6 (arrowhead) (Supplementary Movie 2 at http://www.asnneuro.org/an/006/an006e135add.htm).

We evaluated by immunofluoresecent staining and confocal microscopy whether the ensheathment of the axon resulted in the expression of nodal or paranodal proteins indicative of compact myelin from co-cultures maintained for 14 days in 0.77 nM Gas6 (55 ng/ml) and 2.8 nM Gas6 (200 ng/ml). Co-cultures were incubated with antibodies against NF, MBP and a nodal/paranodal protein including caspr, ankyrinG, claudin11, NaV_1.6_, and neurofascin 155 and 186 and examined by confocal microscopy. We did not find localization of nodal or paranodal proteins immediately adjacent to MBP^+^ OL processes ensheathing NF^+^ axons in the vast majority of co-cultures examined. Based on these finding we conclude that within our *in vitro* co-cultures, Gas6 enhances axonal ensheathment and the formation of noncompact myelin but does not drive the formation of noncompact myelin to compact myelin formation. The neurofascin antibody (NeuromAb) showed nodal formation at a few MBP^+^ axons in coverslips examined (data not shown).

### Electron microscopic analysis confirms OL processes in contact with and ensheathing axons

**Figure 6:**
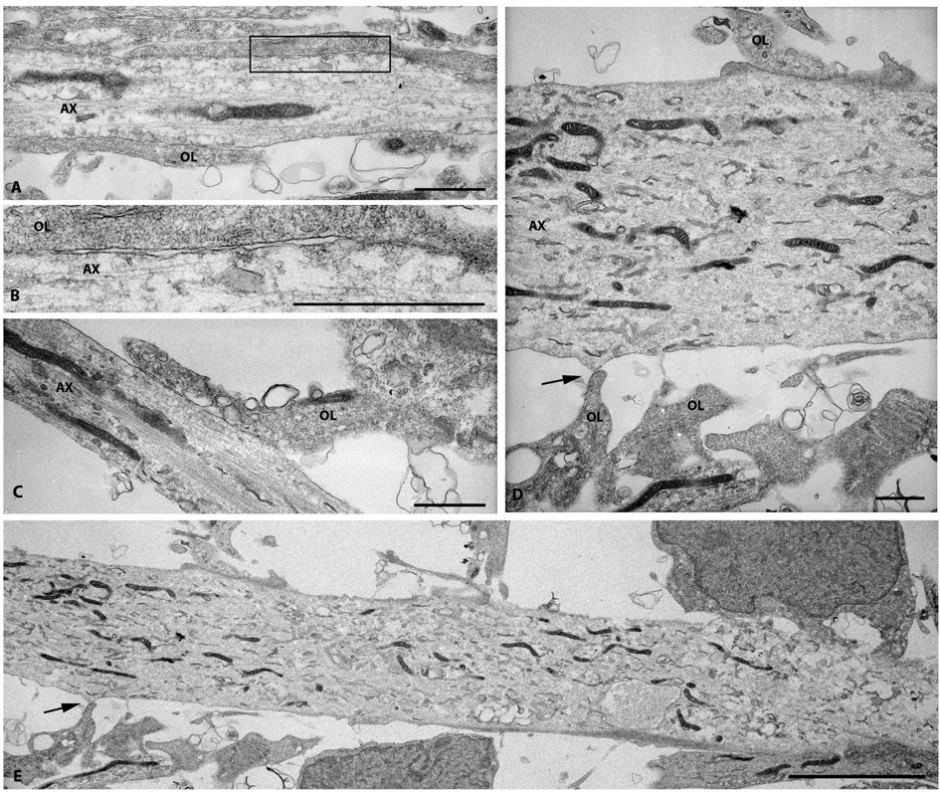
Multiple OL/axonal contacts are observed in Gas6-treated co-cultures A co-culture maintained for 14 days in defined medium plus Gas6 was embedded in Epon, and uranyl-stained and 1-μm sections were examined by EM. Examination of grids found fields containing axons with multiple oligodendrocytes. Scale bars in (**A**–**D**) are 1 μm; the scale bar in (**E**) is 0.5 μm.

Immunofluoresecent staining and microscopy combined with Volocity 3D software showed MBP^+^ processes wrap axons in co-cultures. We examined OL process/axon interactions and the extent of wrapping by electron microscopy. Figure 6 shows that multiple oligodendrocyte processes are in contact with axons. In addition, axonal–OL interactions suggest that axonal ensheathment by OL processes requires a critical axonal diameter. Axons (AX) shown in Figures 6A, 6(C), 6(D) and 6(E) are ~5 μm in diameter. We did not observe contacts between OLs and small diameter axons ≤1 μm. As seen in Figure 6(A), we observed numerous elongated OL membranes forming contacts with an axon. The box is enlarged to show multiple OL–axon membrane contacts (Figure 6B). An OL process along an axon is shown in Figure 6(C). In Figure 6(D) multiple OLs near and contacting an axon are observed. Figure 6(E) illustrates the axon in Figure 6(D) at low magnification to indicate the OL processes in the upper-left corner contacting the axon at three sites consistent with our light microscopic data.

**Figure 7:**
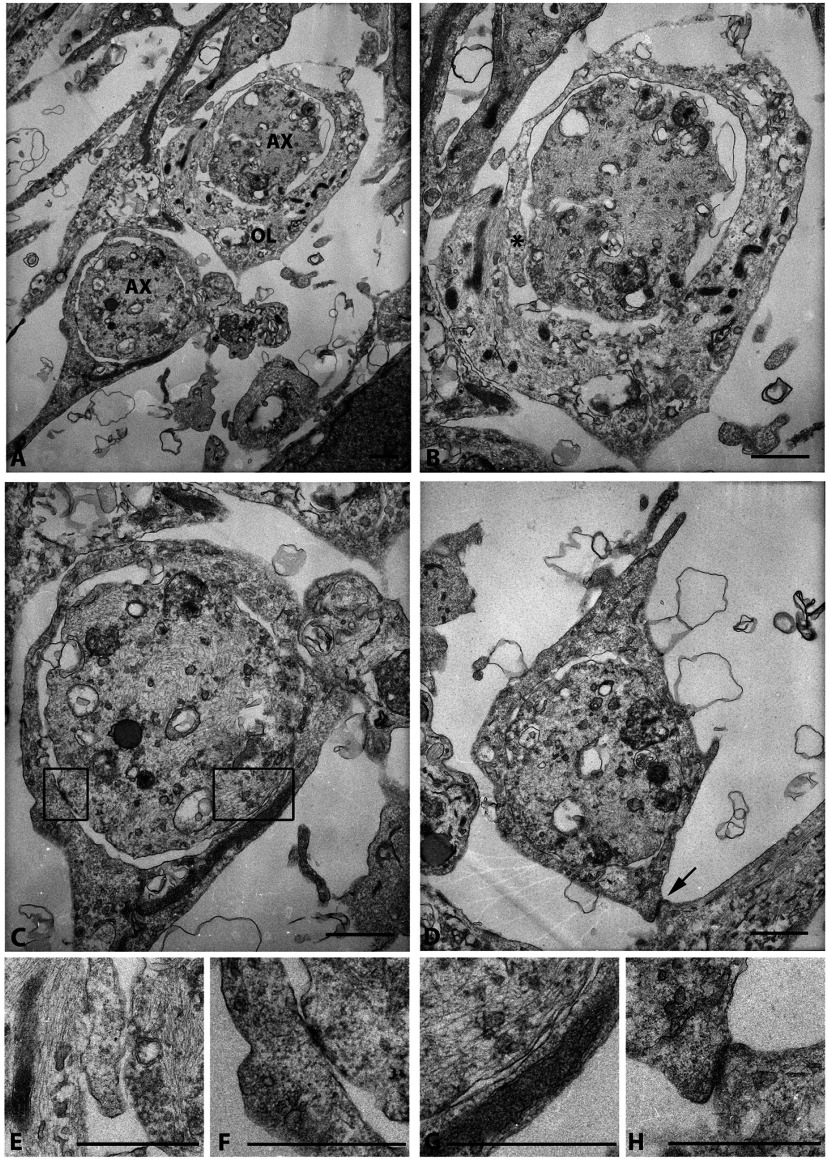
Visualization of wrapping of axons in human co-cultures in the presence of Gas6 (2.6 nM) (**A**) Two axons (AX) wrapped in myelin membrane. (**B**) Upper axon from (**A**) shows wrapping of one myelin membrane encircling the other (*); higher magnification * is shown in (**E**). (**C**) Lower axon from (**A**) shows OL membrane wrapping and multiple contacts/adhesions between the axonal membrane and the OL membrane; higher magnifications of the contacts are shown in (**F**) and (**G**). (**D**) OL membrane wrapping an axon, and contacting an additional membrane; higher magnification is shown in (**H**). Scale bars are 1 μm.

In addition to observing multiple OL–axon membrane contacts we observed OL processes wrapping the axon supporting our light microscopic data. The majority of the wraps observed were one to two wraps of myelin membrane ensheathing the axon and with these co-cultures we did not observe compact myelin. Figures 7(A)–7(D) shows representative axons wrapped in myelin membrane. Interestingly, we observed in several grids with OL membrane wrapping and multiple contacts/adhesions between the axonal membrane and the OL membrane; see asterisk in Figure 7(B) and higher magnifications of the contacts in Figures 7(F) and 7(G). Additionally, as observed in Figure 7(D), we observed an OL membrane wrapping an axon, as well as contacting an additional axonal membrane; higher magnification in Figure 7(H). These data show that human fetal OL/DRG co-cultures generate non-compact myelin that is able to contact, elongate, form physical contacts, and ensheath axons. However, ultrastructural analysis failed to observe compact myelin in co-cultures maintained in Gas6.

## DISCUSSION

Myelination of axons is a highly regulated process relying upon neuronal activity, adhesion, extracellular ligands, secreted molecules and receptor engagement on OLs and axons (Emery, 2010; Piaton et al., 2010). With the exception of the axonal initial segment, nodes and termini, the vast majority of axons are ensheathed along their length to maximize axonal conductance velocity. In the vertebrate CNS, myelin is generated by elongated and flattened OL processes which ensheath axons in multiple layers of lipid-rich plasma membrane. Mature myelin is composed of both noncompact and compact myelin consisting of lipids and several specialized proteins synthesized by the OL. MBP, a 14–22.5 kDa protein, is one of the most abundant proteins expressed in myelin. MBP is highly charged and interacts with the cytoplasmic side of the membrane serving as a molecular sieve to allow, as well as restrict, select proteins to be incorporated into the myelin sheath (Aggarwal et al., 2011). Based on the important role of MBP in myelination, we developed a human co-culture model system to enable us to examine the effect of Gas6 and other signaling molecules on MBP expression and myelination.

We have determined that Gas6 enhanced ensheathment of axons by MBP^+^ processes in human co-cultures. In earlier studies using microarray analysis and RT–PCR, we determined that transcripts for PI3K, Axl and Mer were expressed at very high levels in O4^+^ human OLs when myelination was ongoing in fetal spinal cord. Our current data support these findings and suggest a role for Gas6 signaling in ensheathment of the axon by myelin membranes generating noncompact myelin. Gas6 is detected in CSF (Sainaghi et al., 2008), and is expressed in and secreted by motor neurons and large neurons of the DRG (Li et al., 1996; Allen et al., 1999). Gas6 and its receptors are expressed widely in the CNS (Crosier and Crosier, 1997; Avanzi et al., 1998; Allen et al., 1999; Prieto et al., 2000). *In situ* hybridization studies performed in rat CNS demonstrated that members of the TAM family are expressed on neurons and in white matter during myelination (Allen et al., 1999; Prieto et al., 2000) supporting our finding that Gas6/receptor interaction may enhance adhesion and ensheathment between axons and OLs during myelination, where high levels of both ligand and receptor are observed (Prieto et al., 2000).

Gas6 activates Axl, Tyro3, and Mer receptor tyrosine kinases in a concentration-dependent manner. While we have no evidence that Axl is the receptor to which Gas6 is signaling to in our co-culture model, Axl is preferentially activated by Gas6 and is expressed during myelination (Prieto et al., 2000). Also, Axl is required for Gas6-enhanced OL survival during tumor ncerosis factor (TNF) challenge (Shankar et al., 2006). Axl contains two binding sites for Gas6; Tyro3 and Mer each have one binding site. In the developing CNS, Axl down-regulation results in increased Tyro3 expression, but both continue to be expressed in the mature CNS. Ligand/receptor engagement results in kinase activation and autophosphorylation at three tyrosine residues within the cytoplasmic domain, and the recruitment of signaling molecules such as Grb2 (pYXNX) and PI3K (pYXXM) via the p85 subunit. Gas6 activation and recruitment of Grb2 can activate MAPK and ERK (Fridell et al., 1996). Whether the Grb2 binding domain and the PI3K domain can be engaged simultaneously or occurs sequentially is yet to be determined. In survival studies we determined that the p85 subunit of PI3K bound to the pYXXM site, and recruited active PI3K (p85p110). Also, we determined that Akt activation at the membrane occurred within 5 min of Gas6 treatment (Weinger et al., 2008). The ability of TAM receptors to recruit Grb2 and signal to ERK kinase is intriguing as ERK signaling has been implicated in axonal ensheathment (Furusho et al., 2012). It is likely that there is redundancy in the various growth factors and signaling molecules and several may be required to achieve ensheathment and extensive compact myelin formation *in vitro* and *in vivo*.

Gas6 was shown to enhance survival, and maturation of PNS and CNS cells including neurons, OLs and Schwann cells (Li et al., 1996; Shankar et al., 2003, 2006). In this study, we have observed positive effects on myelination with Gas6 concentrations of 0.77–5.6 nM. By ELISA, we determined that co-cultures maintained in the absence of Gas6 express ~852 pg/ml Gas6 (~12 pM) into the medium, although we cannot directly equate this to the local Gas6 concentration immediately following secretion by the neuron. Gas6 concentrations in the CSF from patients with various diagnoses including multiple sclerosis, stroke, amyotrophic lateral sclerosis, headache, psychiatric conditions simulating neurological diseases, otologic dizziness, Guillain–Barré syndrome (GBS) or chronic inflammatory demyelinating polyneuropathy were 5.6–11.5 ng/ml (Sainaghi et al., 2008, 2013).

For all our studies, we have only used recombinant human Gas6 that was grown in vitamin K-containing medium and purified at Amgen. The resulting Gas6 is γ-carboxylated, measurable in an ELISA assay, and is able to phosphorylate Axl and Tyro3 (Brown et al., 2012). We have determined that several of the commercially available recombinant Gas6 do not contain the Gla domain, are not grown in the presence of vitamin K, and are not tested for activity, only in a proliferative response. Recombinant mouse Gas6 (R&D Systems) has extremely low activity in *in vitro* kinase assays where minimal Tyro3 was autophosphorylated (personal communication, A. Prieto). We do not know whether the Gla domain of Gas6 is required for the ensheathment of axons by Gas6, and we have not found any published studies that demonstrate that Gas6 minus the Gla domain is active *in vitro* or *in vivo*. We know that the Axl-Fc decoy is able to block the beneficial effects of Amgen's Gas6 in primary OL cultures (Shankar et al., 2006).

Factors differentially expressed by axons, OPCs, and myelinating OLs rely on activation of promyelinating signaling molecules as well as the suppression of extracellular inhibitory signals. We examined the ability of the human DRGs and OPCs to support myelination at several gestational ages. OPCs isolated from brains at 14 gestational weeks and younger never synthesized MBP^+^ OLs when co-cultured with DRGs from early or late gestational ages. In two independent experiments, we determined that OPCs from 14 gestational weeks or younger did not mature to MBP^+^ OLs in co-culture even when the co-cultured were maintained for 21 days. We determined that the DRG gestational week is less critical than the OPC gestational age as 15 gestational week DRGs could support myelination of 16 gestational week OPCs. OPCs from 19 gestational weeks yield the highest number of MBP^+^ OLs with some co-cultures containing as many as 470 MBP^+^ OLs/12 mm coverslip. However, the total percentage of MBP^+^ OLs relative to the initial 100000 OPCs plated at day 0 was less that 1%.

We determined that administration of Gas6 by mini-pump directly to the corpus callosum enhanced re-myelination following cuprizone-induced demyelination (Tsiperson et al., 2010). Additionally, Gas6-knockout mice had a delay in remyelination during the recovery phase following cuprizone withdrawal suggesting that Gas6 promotes myelination (Binder et al., 2011). Additional growth factors such as BDNF enhance MBP^+^ expression (VonDran et al., 2010, 2011) in murine oligodendrocytes and in rodent co-cultures (Prieto et al., 1999, 2000; Cui et al., 2010). IGF1 increases myelin in transgenic mice and myelination is altered in IGF1 null mice (Carson et al., 1993; Ye et al., 2002). We determined that BDNF, IGF and Gas6 enhanced ensheathment of axons in human co-culture, and EM confirmed our immunoflorescent staining and confocal microscopy findings. Our studies demonstrated that multiple OLs can contact a single axon, and multiple MBP^+^ processes from single OLs can ensheath a single axon, or multiple axons. The nature of the proteins contained at the “adhesion contact” observed between the OL membrane and the axon by EM requires further analysis. A recent paper suggest that both the adhesive properties of proteolipid protein and the loss of sialic acid residues from the cell surface during myelinogenesis aid in myelin membrane adhesion and the compaction of CNS myelin (Bakhti et al., 2013).

Finally, we examined by EM whether there is a preferential axonal diameter for initial myelination in our human co-cultures. We determined that the majority of myelinated axons had a mean axon diameter of ~5 μm. It is possible that the limited number of axonal wraps observed in our co-cultures is the result of variability in the diameter of the axons. Small axons tend not be myelinated, and larger or swollen axons are more difficult to myelinate. We determined that aged DRG explants maintained for longer than 4 weeks did not efficiently support myelination when plated with OPCs in co-culture. Additionally, 1% paraformaldehyde-fixed axons did not support human OPC formation of MBP^+^ myelin membranes. While our studies show that Gas6 supports wrapping of noncompact myelin membranes in a human co-culture system, many aspects of myelin biology are still unresolved. Future studies will explore additional pathways to determine whether activators and inhibitors affect wrapping, and whether Gas6 can synergize with signal transduction pathways to enhance compact myelin formation. Thus, for successful myelination to occur, intrinsic and extrinsic factors must be co-ordinately regulated both at the axon and at the OL. The co-ordinated integration of multiple intrinsic signaling pathways determines when differentiation and maturation in the human CNS occurs. Our methodological analyses for the establishment of human DRG/OL co-cultures provide a foundation for future *in vitro* studies of human myelination. Furthermore, our data demonstrate that IGF1 and BDNF enhance the expression of mature myelin proteins, including MAG and MBP, and supplementation with Gas6 results in OL processes extending myelin membranes that augment axonal ensheathment. Growth factors including insulin, BDNF, IGF1, thyroid hormone and Gas6 are among the ligands that, through receptor engagement and downstream signaling by multiple kinases, regulate OL differentiation and myelination. Clearly, the complex interactions necessary to obtain axonal ensheathment, noncompact myelin, and compact myelin formation will require cross-talk and integration of multiple signaling pathways. The MAPK/ERK, the cAMP pathway, PI3K/Akt and mammalian target of rapamycin (mTOR) signaling pathways have all been implicated in myelination (Hannila and Filbin, 2008; Nave, 2010; Wood et al., 2013)

## Online data

Supporting Movie

Supporting Movie
